# *Stenotrophomonas* in diversified cropping systems: friend or foe?

**DOI:** 10.3389/fmicb.2023.1214680

**Published:** 2023-08-03

**Authors:** Abhishek Kumar, Lellapalli Rithesh, Vikash Kumar, Nikhil Raghuvanshi, Kautilya Chaudhary, Abhineet, Abhay K. Pandey

**Affiliations:** ^1^Department of Plant Pathology, Chaudhary Charan Singh Haryana Agricultural University, Hisar, Haryana, India; ^2^Department of Agriculture, Maharishi Markandeshwar (Deemed to be University), Mullana, Ambala, Haryana, India; ^3^Department of Plant Pathology, Kerala Agricultural University, Thiruvananthapuram, Kerala, India; ^4^Faculty of Agricultural Sciences, Institute of Applied Sciences & Humanities, GLA University, Mathura, Uttar Pradesh, India; ^5^Department of Agronomy, Institute of Agriculture and Natural Science, Deen Dayal Upadhyaya Gorakhpur University, Gorakhpur, Uttar Pradesh, India; ^6^Department of Agronomy, Chaudhary Charan Singh Haryana Agricultural University Hisar, Hisar, Haryana, India; ^7^Department of Agriculture, Integral Institute of Agricultural Sciences & Technology, Integral University, Lucknow, Uttar Pradesh, India; ^8^Department of Mycology & Microbiology, Tea Research Association, North Bengal Regional R&D Center, Nagrakata, West Bengal, India

**Keywords:** crop protection, ecofriendly, ecosystem, organic, osmoprotective, PGPR

## Abstract

In the current scenario, the use of synthetic fertilizers is at its peak, which is an expensive affair, possesses harmful effects to the environment, negatively affecting soil fertility and beneficial soil microfauna as well as human health. Because of this, the demand for natural, chemical-free, and organic foods is increasing day by day. Therefore, in the present circumstances use of biofertilizers for plant growth-promotion and microbe-based biopesticides against biotic stresses are alternative options to reduce the risk of both synthetic fertilizers and pesticides. The plant growth promoting rhizobacteria (PGPR) and microbial biocontrol agents are ecologically safe and effective. Owning their beneficial properties on plant systems without harming the ecosystem, they are catching the widespread interest of researchers, agriculturists, and industrialists. In this context, the genus *Stenotrophomonas* is an emerging potential source of both biofertilizer and biopesticide. This genus is particularly known for producing osmoprotective substances which play a key role in cellular functions, i.e., DNA replication, DNA-protein interactions, and cellular metabolism to regulate the osmotic balance, and also acts as effective stabilizers of enzymes. Moreover, few species of this genus are disease causing agents in humans that is why; it has become an emerging field of research in the present scenario. In the past, many studies were conducted on exploring the different applications of *Stenotrophomonas* in various fields, however, further researches are required to explore the various functions of *Stenotrophomonas* in plant growth promotion and management of pests and diseases under diverse growth conditions and to demonstrate its interaction with plant and soil systems. The present review discusses various plant growth and biocontrol attributes of the genus *Stenotrophomonas* in various food crops along with knowledge gaps. Additionally, the potential risks and challenges associated with the use of *Stenotrophomonas* in agriculture systems have also been discussed along with a call for further research in this area.

## Introduction

In recent years, the harmful effects of pesticides and synthetic fertilizers on humans, animals as well as on the whole ecosystem have led to the expansion of novel beneficial microbes. There are likely many undiscovered microorganisms in unexplored plants and soils that may play a crucial role in promoting plant growth through their various activities. The bacterial genus *Stenotrophomonas* is referred to as a potential PGPR with advantageous effects because of its capacity to produce siderophores, the ability to solubilize phosphate, and the generation of phytohormones and spermidine (Ulrich et al., 2021). This genus belongs to the family Xanthomonadaceae as an emended description of the Lysobacteraceae family (Cutiño-Jiménez et al., 2020). The Lysobacteraceae (Xanthomonadaceae) family covers a diverse group of bacteria, which includes *Pseudoxanthomonas, Stenotrophomonas*, *Xanthomonas*, and *Xylella*, these are closely related bacterial genera that form a phylogroup referred to as XSXP (Bansal et al., 2021). The Xanthomonadaceae family also contains plant pathogenic bacteria namely *Xanthomonas* and *Xylella,* which are reported to cause economic losses in several crops (Parte, 2018). On the other hand, this family also included the PGPR including *Pseudomonas geniculata* and *S. rhizophilia* with medical, environmental, and biotechnological significance (Cutiño-Jiménez et al., 2020). The *Stenotrophomonas maltophilia* was earlier described as *Pseudomonas maltophilia* in the year 1961 (An and Berg, 2018; Wang et al., 2018a,b).

*Stenotrophomonas* species are Gram-negative and associated with wide a range of habitats, including animals as well as plant hosts (Hayward et al., 2010). Additionally, this bacterium is cosmopolitan and ubiquitous that found in an environmental habitat range, including extreme ones, although naturally it is associated with plant’s rhizosphere and mainly contributed to the elemental cycling of sulphur and nitrogen, and also degrades complex compounds and pollutants, and promotes the growth of plants and their health (An and Berg, 2018; Pérez-Martínez et al., 2020). Moreover, the bacterium *S. maltophilia* is the first member of this genus which is a predominant species observed in plants, water, soil, animals, and humans (Wang et al., 2018b). *Stenotrophomonas maltophilia* has a sequenced genome with a genome size of approximately 4.8 Mbp with a G + C content of 66.7% (Crossman et al., 2008). The whole genome sequence analysis of *S. indicatrix* BOVIS40 yielded a 4.42 Mb genome size with ~66.4% G + C content (Adeleke et al., 2021). Bansal et al. (2021) suggested that, in light of deep phylotaxonomy genomics findings along with published polyphasic data, XSXP phylogroup warrants reunification and need to consider *Xylella, Stenotrophomonas,* and *Pseudoxanthomonas* as synonyms of *Xanthomonas*.

**Figure 1:**
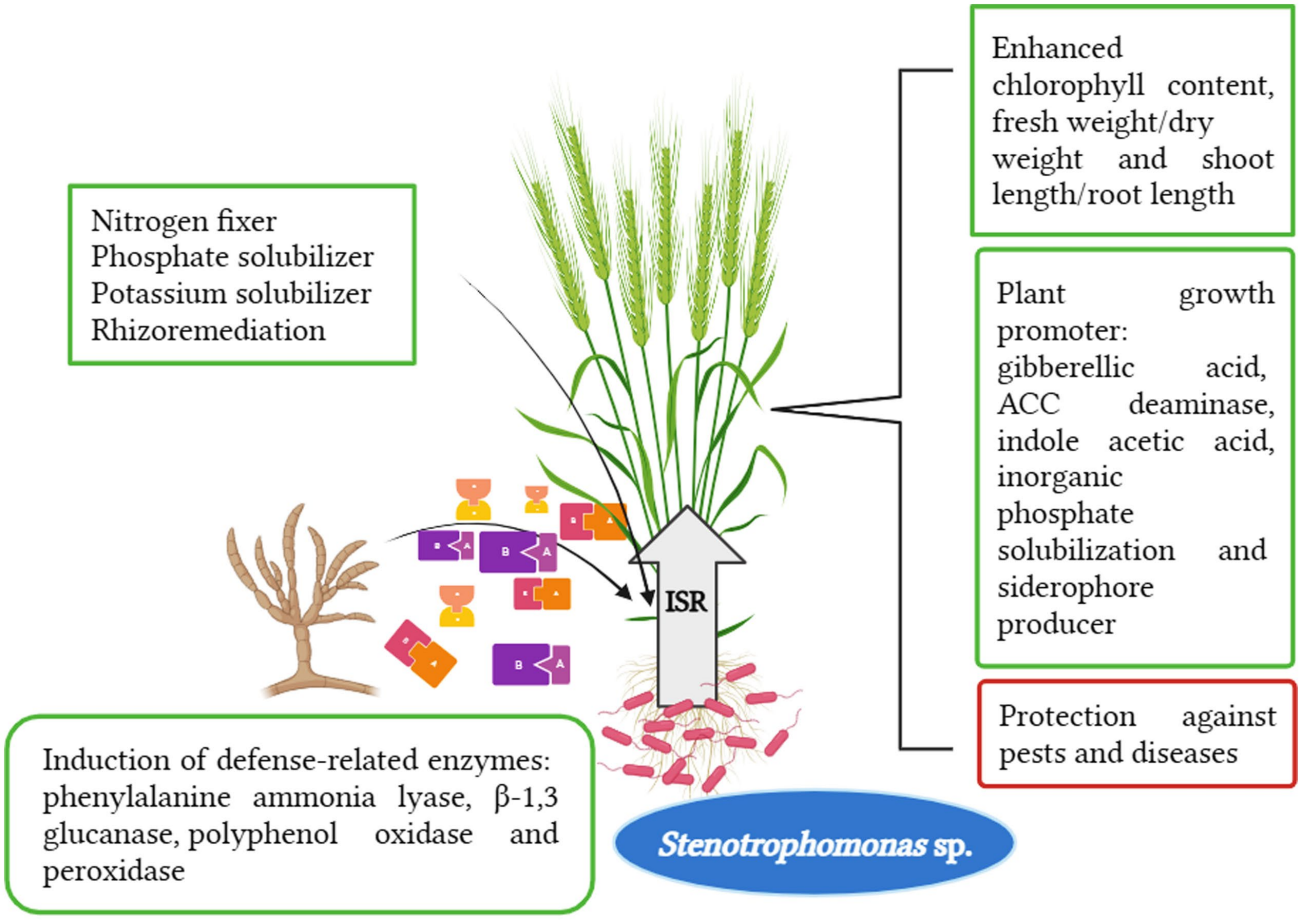
Multi-fold action of *Stenotrophomonas* spp. in plant system.

Many researchers reported the benefits of the genus *Stenotrophomonas* for plant systems (Figure 1). The genus *Stenotrophomonas* colonizes extreme manmade niches in space shuttles, hospitals, and clean rooms (An and Berg, 2018). *Stenotrophomonas* bacteria are becoming more researchable because of their potential use as effective bioinoculants for promoting plant growth and managing several diseases of food crops. This aspect is of growing biotechnological interest (Ulrich et al., 2021). Nowadays, this genus has become a research opportunity as a multidrug-resistant human pathogen, which does not commonly infect healthy humans but it may be associated with high morbidity and mortality in severely immunocompromised and debilitated patients by causing several infectious diseases (Flores-Treviño et al., 2019). These bacteria can also be recovered from polymicrobial infections, most especially from the respiratory tract (lungs) of cystic fibrosis patients (Brooke, 2014). Additionally, it has biodefense capacity against plant pathogenic fungi and bacteria as well as resistance to biotic and abiotic stress, anti-quorum sensing, and anti-biofilm bioactivities (Ulrich et al., 2021). The closely related *S. rhizophilia* provides a substitute for biotechnological applications without any harm to human health (An and Berg, 2018).

The role of *Stenotrophomonas* in human infectious diseases has been discussed previously (Berg and Martinez, 2015; Anđelković et al., 2019; Gil-Gil et al., 2020; Dadashi et al., 2023), but to date, there have been no reviews on its significance of plant disease management and plant growth promotion. In this review, the growth and biocontrol traits of the genus *Stenotrophomonas* in several food crops, as well as the probable risks and challenges associated with the use of *Stenotrophomonas* in agriculture systems and potential future research, have been discussed.

## Biocontrol potential of the genus *Stenotrophomonas*

### Efficiency against plant pathogens

In recent years, the use of antagonistic microorganisms to manage plant diseases has been regarded as a viable alternative to synthetic fungicides. The effects of beneficial microorganisms on growth improvement and higher disease tolerance have been studied in many cropping systems. Microbial biocontrol agents have been shown to have a high possibility of enhancing the yield of food crops by increasing phosphate solubilization, fixing nitrogen, or restricting diseases (Parnell et al., 2016). The genus *Stenotrophomonas* is used as a potential microbial biocontrol agent against many plant pathogens hampering commercial crops (Mahaffee and Kloepper, 1997; Germida and Siciliano, 2001; Mehnaz et al., 2001; Berg et al., 2002). In addition to their other plant growth-promoting characteristics, *Stenotrophomonas* spp. exhibited antagonistic patterns against pathogenic oomycetes, fungi, bacteria, and insect pests. There are several *Stenotrophomonas* spp. that may interact well with plants, especially *S. maltophilia* and *S. rhizophilia* (Ryan et al., 2009). However, still, no report is available on the plant pathogenic nature of *Stenotrophomonas* and its related species.

According to de Oliveira-Garcia et al. (2003), *S. maltophilia* produces various kinds of pili that are involved in the development of complex biofilms as well as adherence to surfaces. The potential of *S. maltophilia* to interact with other microbes on the plant’s surface may be influenced by both adhesion and biofilm formation (Elvers et al., 2001). *S. maltophilia* strains has *S. maltophilia* fimbriae 1 (SMF1) fimbriae, which is made up of fimbrin subunits that are significantly similar to numerous pathogenic *Escherichia coli* and the *Pseudomonas aeruginosa* fimbria in terms of amino-terminal of amino acid sequence. The genome of *S. maltophilia* strains includes genes that encode type I and type IV pili, type I pili involved in adhesion and the early phases of biofilm formation. Type IV pili are also involved in adherence, auto aggregation, twitching motility, and biofilm formation. This might imply that invasion strategies of *Stenotrophomonas* spp. in plants form biofilms and exhibit twitching motility. *S. maltophilia* has the mannose-6-phosphate *(Man)* gene and lipopolysaccharide/exopolysaccharide-coupled biosynthetic genes (*rmlA*, *rmlC*, and *xanB*), which encode enzymes involved in the synthesis of lipopolysaccharides (LPS) and exopolysaccharides (Huang et al., 2006).

The biocontrol ability of *Stenotrophomonas* spp. has been proved in numerous crops. Through several mechanisms, these bacteria suppress the growth of phytopathogens. The novel antifungal substances maltophilin (Jakobi et al., 1996) and xanthobaccin (Nakayama et al., 1999) have been identified, and the majority of *S. maltophilia* isolates exhibit antifungal activity against various pathogens *in vitro* (Minkwitz and Berg, 2001). The strains of *S. maltophilia* generate a wide range of proteases, chitinases, glucanases, RNases, DNases, lipases, and laccases, and they have an incredibly high hydrolytic potential (Tandavanitj et al., 1989; Galai et al., 2009). The biocontrol capability of *S. maltophilia* includes both chitinolytic and proteolytic activities. Through the breakage of fungal cell walls, chitinases may protect plants against phytopathogens, but they may also play a part in activating induced defense systems (Mastretta et al., 2006). It is necessary to determine the precise roles played by the several exoenzymes involved in the antagonistic activity of *Stenotrophomonas* spp., thus making it possible that additional bacterial products may similarly control plant disease by inducing plant defenses.

The competition for iron is another essential factor in preventing pathogens. Siderophores produced by various pathogens such as ferrichrome, which is produced by several fungi, including the phytopathogenic *Ustilago maydis* can be efficiently captured by *Stenotrophomonas* (Ardon et al., 1997). The sequenced *Stenotrophomonas* spp. genomes feature large amounts of volatile organic compounds (VOCs), outer membrane proteins, and TonB-dependent receptors (TBDRs). Moreover, *Stenotrophomonas* spp. may release VOCs that act as inter- and intracellular communication signals and adversely influence the development of several pathogens (Wheatley, 2002). Several distinct VOCs produced by *S. maltophilia* and *S. rhizophila* inhibited the development of *Rhizoctonia solani*, which causes severe damage to economically significant crops and trees worldwide. Phenylethanol and dodecanal are the VOCs that have been identified in this study (Kai et al., 2007). However, the specific mechanism by which these secondary metabolites affect the pathogen is unknown and needs further research.

Further, S*tenotrophomonas* spp. release many hydrolytic enzymes that are important in the inhibition of plant diseases, including lipases, chitinases, RNAses, DNAses, and proteases (Berg et al., 2010). *S. maltophilia* MB9, a marine-isolated strain, was successful in preventing large numbers of phytopathogens including *R. solani, F. oxysporum,* and *Curvularia* sp. by producing dodecanoic acid, a broad-spectrum antifungal antibiotic (John and Thangavel, 2017). In a dual culture laboratory study, *Stenotrophomonas* spp. displayed significant zones of inhibition against *R. solani, Verticillium dahlia,* and *Sclerotinia sclerotiorum* (Wolf et al., 2002). *S. maltophilia* strains obtained from the rhizosphere of the brinjal crop in Egypt demonstrated inhibitory effects against *Ralstonia solanacearum*, a potato brown rot pathogen. The isolates were useful for decreasing symptoms in potatoes grown in soil in addition to being beneficial *in vitro* (Messiha et al., 2007).

According to Schmidt et al. (2012), a *S. rhizophila* strain (DSM14405T) promoted plant development by changing the rhizosphere microbiome. It was observed that the strain was successful in colonizing both the root and the shoots of cotton and sweet pepper. Based on molecular profiling using single-strand conformation polymorphism (SSCP), the rhizosphere fungal microbiome appears to be affected by *S. rhizophila* DSM14405T. In another study, *S. maltophilia* C3-derived chitinase inhibited conidial development and germ tube extension in *Bipolaris sorokiniana* (Zhang et al., 2001). Amino acid sequencing, polyacrylamide gel electrophoresis, and purification indicated that the isolate produced at least two distinct chitinases with potential antifungal activity (Zhang et al., 2001). Kobayashi et al. (2002) isolated a protein and the responsible gene from *S. maltophilia* 34S1 having chitinolytic and antifungal activities. The release of elicitor molecules by hydrolytic enzymes, in particular, contributes to the activation of plant defense systems in addition to the degradation of pathogenic cell structures. In the other study treatment of wheat plants with *S. maltophilia* SPB-9 during the *in vivo* experiment led to a rise in defense enzymes (Singh and Jha, 2017).

There have also been reports of *Stenotrophomonas* species developing increased disease resistance to plant pathogenic viruses. *S. maltophilia* HW2, which was isolated from cucumber, improved the plant’s resistance to the cucumber green mottled mosaic virus. HW2 application on cucumber may slow down virus replication and prevent the expression of viral protein genes. It also induced the expression of antioxidant enzyme genes and defense-related genes (Li et al., 2016). The most recent research on the biocontrol effectiveness of *Stenotrophomonas* species used against different plant diseases is summarized in Table 1. However, further research is needed to fully understand the mechanisms of action and to optimize (toxicity, formulation, consortium development) the use of this bacterium in agriculture.

**Table 1:** Biocontrol efficiency of *Stenotrophomonas* spp. against plant pathogens.

*Stenotrophomonas* spp.	*Pathogen*	Hosts	Reference
*S. rhizophila*	*Colletotrichum gloeosporioides*	Mango	Reyes-Perez et al. (2019)
*S. maltophilia* E38	*Ralstonia solanacearum*	Tobacco	Li et al. (2023)
*S. maltophilia* CR71	*Botrytis cinerea*	Tomato	Rojas-Solís et al. (2018)
*S. maltophilia* B8	*Fusarium oxysporum* f.sp. *cepae*	Garlic	Dewi et al. (2023)
*S. maltophilia* UN1512	*Colletotrichum nymphaeae*	Strawberry	Alijani et al. (2020)
*S. rhizophila*	*Fusarium proliferatum*	Muskmelon	Rivas-Garcia et al. (2018)
*S. maltophilia* PPB3	*Sclerotium rolfsii*	Tomato	Sultana and Motaher Hossain (2022)
*Stenotrophomonas* sp. BHU-S7 (AgNPs)	*S. rolfsii*	chickpea	Mishra et al. (2017)
*S. rhizophila*	*Leptosphaeria maculans*, *Leptosphaeria biglobosa*	Rapeseed	Schmidt et al. (2021)
*S. maltophilia* UPMKH2	*Pyricularia oryzae*	Rice	Badri Fariman et al. (2022)
*S. maltophilia*	*Magnaporthe grisea*	Rice	Etesami and Alikhani (2016)
*S. rhizophila*	*Fusarium oxysporum*	Cucumber	Wang et al. (2022)
*Stenotrophomonas* sp. TRM2	*Bipolaris sorokiniana*	Wheat	Villa-Rodríguez et al. (2019)
*S. rhizophila* KM01, KM02	*Pythium ultimum*	Chilli	Lara-Capistran et al. (2020)
*S. maltophilia*	*Colletotrichum musae*	Banana	Damasceno et al. (2019)
*S. rhizophila* 88bfp	*Athelia rolfsii*	Turfgrass	Ünal et al. (2019)
*S. chelatiphaga*	*Pseudomonas tolaasii*, *Ewingella americana*	Button mushroom	Aslani et al. (2018)
*S. maltophilia* S23, S24, S26 and S28	*Fusarium oxysporum* f. sp. *lycopersici*	Tomato	Aydi-Ben-Abdallah et al. (2020)
*Stenotrophomonas* sp. S81-1	*Ganoderma boninense*	Oil palm	Fadly et al. (2021)
*S. maltophilia* JVB5	Various pathogens	Sunflower	Adeleke et al. (2022)
*Stenotrophomonas* sp. P7T6–4	*Rhizoctonia solani*	Tomato	Hadi et al. (2020)
*S. rhizophila* 88bfp	*Fusarium cerealis*	Turfgrass	Senocak et al. (2020)
*S. maltophilia* TD 1	*Fusarium solani*	Citrus	Ezrari et al. (2021)
*Stenotrophomonas* sp. AG3	*Macrophomina phaseolina*	soybean	Santos et al. (2021)
*S. rhizophila*	*Xylella fastidiosa*	Olive	Mourou et al. (2022)
*S. maltophilia* strain A1w2	*Xanthomonas oryzae* pv. *oryzae*	Rice	Rahma et al. (2022)
*S. maltophilia* Sg3	*Cucumber Mosaic Virus*	Tobacco	Khalimi et al. (2020)

### Entomopathogenic effect against insect pests

Plant defense against attack by insect pests is associated with a number of phytohormones. Jasmonic acid (JA) is the main phytohormone that supports plant defense against insect pests (War et al., 2012). According to research by Pangesti et al. (2015), the JA signaling pathway is critical for *Arabidopsis thaliana* rhizobacteria-triggered ISR against *Mamestra brassicae* (cabbage moth). It is well known that plants need proteinase inhibitors (PIs) in order to protect themselves against insect pests. Studies revealed that PGPR-mediated ISR is associated with elevated expression of plant responsive genes that encode for PIs and elevated activity of defense-related enzymes (Harun-Or-Rashid and Chung, 2017). In plants that have received the PGPR treatment, defense responses may be generated more rapidly in the case of a pest attack. Inoculating tobacco plants with *S. rhizophilia* may induce JA accumulation and increase the transcript level of JA sensitive genes, resulting in the induction of systemic resistance in tobacco plants against *Spodoptera litura* (tobacco cutworm) (Ling et al., 2022). The biocontrol ability of *Stenotrophomonas* spp. against insect pests is less recorded and further research on integrated management of serious insect pests is required. However, the summary of recent research on *Stenotrophomonas* spp. biocontrol efficiency applied against insect and nematode pests is provided in Table 2. Further research on *Stenotrophomonas* spp. against insects is needed to fully understand its capabilities and limitations. However, the promising results of recent studies suggest that *Stenotrophomonas* may be a useful lever in integrated pest management for a variety of crops (Table 3).

**Table 2:** Entomopathogenic effect of *Stenotrophomonas* spp. against insect pests.

*Stenotrophomonas* spp.	Insect	*Scientific name*	Reference
*S. tumulicola* T5916-2-1b	Aphids	*Aphis punicae*, *Aphis illinoisensis*	Baazeem et al. (2022)
*S. maltophilia*	Termites	*Coptotermes heimi*, *Heterotermes indicola*	Jabeen et al. (2018)
*S. maltophilia* W 2–7	Root-Knot Nematode	*Meloidogyne* spp.	Vishnu and Nisha (2020)
*S. rhizophila*	Tobacco cutworm	*Spodoptera litura*	Ling et al. (2022)
*S. maltophilia*	Potato beetle	*Leptinotarsa decemlineata*	Aktas et al. (2022)

**Table 3:** The effect of *Stenotrophomonas* spp. on host plant.

Host/ Source	Strain	Homology to the reference strain	Accession no.	Colonization status	Action	Reference
Rhizosphere of tomato plants	Two oxalotrophic strains (OxA and OxB)	*S. maltophilia*	-	Endophytically	Strain OxA and OxB protected host from the damage caused by high doses of oxalic acid Protected host from *S. sclerotiorum* and *B. cinerea* infections. Moreover, Callose deposition induced by OxA and OxB was required for protection against phytopathogens Inoculation of theses bacteria induced the production of phenolic compounds and the expression of PR-1 Both isolates exerted a protective effect against fungal pathogens in *Arabidopsis* mutants affected in the synthesis pathway of salicylic acid (sid2-2) and jasmonate perception (coi1) Moreover, *B. cinerea* and *S. sclerotiorum* mycelial growth was reduced in culture media containing cell wall polysaccharides from leaves inoculated with each bacterial strain	Marina et al. (2019)
*Equisetum arvense*	ES2	*S. maltophilia*	KY486848	Endophytically	Greatest ability to synthesize indole-3-acetic acid (IAA)-like compounds, Highest metabolic activity based on the Biolog GEN III test.	Woźniak et al. (2019)
*Zea mays*	ZR5	*Stenotrophomonas* sp.	KY486808			
*Arctium lappa*	AR4	*S. maltophilia*	KY486847			
*Pistacia atlan-tica* L.	Sm25	*S. maltophilia* IAM12423^T^	KU693275	Endophytically	Siderophore production Plant growth promotion	Etminani and Harighi (2018)
	Sm97	*S. maltophilia* IAM12423^T^	KU693276			
Nodule-associated bacteria (NAB) were isolated from wild legume nodules	LXo13	*Stenotrophomonas* sp.	–	–	The most frequent PGP activity identified among the strains isolated from wild legumes was IAA synthesis Two bacteria, *Stenotrophomonas* sp.	Tapia-García et al. (2020)
	LX010a					
Rhizospheric isolates	NGB-15	*Stenotrophomonas* sp. strain KG-16-3	LC322228	Rhizospheric	Phosphate solubilization Plant growth promoting activity over the control (increased shoot and root fresh and dry biomass)	Youseif (2018)
	NGB-18	*S. maltophilia* strain E136	LC322229			
Corn Rhizosphere	L,o.INCA-FRr1	*S. rhizophila*	–	Rhizospheric	Ability to perform the FBN, Solubilization of phosphorus, potassium and antagonistic activity against *Fusarium oxysporum*	Pérez-Martínez et al. (2020)
	INCA-FRc24	*S. pavanii*	–			
Aerial parts of poplar	*Stenotrophomonas* strain 169	*Stenotrophomonas* strain Fa6 (AY131216)	CP061204	Endophytically	The root weight of the inoculated plants was three times higher than that of the untreated plants, Showing a significant increase in both root and shoot length of the inoculated *in vitro* plants Tolerance of plants to abiotic stresses Production of siderophores and the ability to mobilize phosphates, as well as the production of the plant growth-promoting substances IAA and polyamines	Ulrich et al. (2021)
11 wild plants (rhizosphere and phyllosphere)	*Stenotrophomonas* sp. *TmP43c*	*S. rhizophila*	–	rhizosphere	Highest number of psychrotolerant strains, Higher phosphate solubilization activity	Vega-Celedón et al. (2021)
Sugarcane rhizosphere	COA2	*S. maltophilia*	MN527324	Rhizospheric	Phosphate solubilizing, Siderophore Production IAA production Ammonia Production ACC production Antagonistic activity Increased the activity of SOD, CAT, PAL, CHI, and GLU enzyme Nitrogen fixative	Singh et al. (2020)
Sunflower root endosphere	SAMN18138830	*S. indicatrix* BOVIS40	JAGENA000000000	Endospheric	Plant growth promoter IAA production Siderophore production Phosphate solubilizing Exopolysaccharide Amylase, Cellulase, Xylanase, Mannanase and Protease enzymes enhancer	Adeleke et al. (2021)
Strawberry leaves	UN1512	*S. maltophilia*	MT448956	Endophytically	Antagonistic effect against *C. nymphaeae* in dual culture Secreted protease, chitinase, pectinase, siderophore, IAA, and gibberellin Produced volatile compounds (Benzothiazole, Cyclooctatetraene-1-carboxaldehyde, Carbonic acid, octadecyl phenyl ester, Benzaldehyde, 2,5-bis, Estragole, Benzaldehyde) Growth promoter	Alijani et al. (2020)
Oil-free soil	SR1	*S. maltophilia-*SR1	MH634684	Rhizospheric	Highest plant growth promoter Upregulated nitrate, nitrate reductase, total nitrogen, and nonenzymatic and enzymatic antioxidants in plants Suppressed oxidative and nitrosative stress Produced indoleacetic acid and ammonia as well as phosphate solubilization	Bashandy et al. (2020)
Tomatillo (*Physalis ixocarpa*) roots	CR71	*S. maltophilia*	MF992168	Endophytically	Promoted the shoot and root length, chlorophyll content, and total fresh weight of tomato Biocontrol of *B. cinerea* through the production of potent volatiles such as dimethyl disulphide	Rojas-Solís et al. (2018)
Salt-resistant *Carex distans* (distant sedge) roots	SRS1	*S. rhizophila* DSM14405	–	Endophytically	Increased the induction of plant genes related to abscisic acid and auxin signaling Enhanced plant growth	Manh Tuong et al. (2022)
*Mucuna utilis* var. *capitata* L. (Safed Kaunch)	RMC6	*S. maltophilia*	HM480495	Endophytically	Antagonistic against *Fusarium udum* Plant growth promoter (IAA, phosphate solubilization and ACC deaminase, siderophore production)	Aeron et al. (2020)
Soil Contaminated by Industrial Effluent	S25	*S. maltophilia*	KY651248	rhizosphere	As-reducing capability Produced Hydrogen Cyanide, Nitrogen Fixation, and Auxin Plant growth promoter	Huda et al. (2022)

## Plant growth promoting activity of *Stenotrophomonas* spp.

Biofertilizers are a reliable source to promote plant growth without harming the soil, plants and environment (Reddy et al., 2020). The plant growth promoting rhizobacteria could be a viable substitute to overcome the load of synthetic fertilizers which are used indiscriminately in the agricultural sector as well as to enhance farmers income because they are cost-effective and durable (Pérez-Martínez et al., 2020). In this context, the genus *Stenotrophomonas is* characterized as promising plant growth promoting bacterium, which are reported as inducers and protectors against biotic and abiotic stresses (Table 3).

In addition to that, the three isolates of the genus *Stenotrophomonas* isolated from healthy tomato plants were able to produce indole-3-acetic acid; two of these strains had phosphate solubilization ability (Ben Abdallah et al., 2018). *S. maltophilia* P9 was isolated from the algal biomass and identified as a potential pectinase producing with biotechnological significance (Sharma and Sharma, 2018). In a study, Woźniak et al. (2019) reported that *Stenotrophomonas* strain ES2 promotes growth under *in vitro* conditions. Seed treated wheat plants with rhizospheric *S. maltophilia* SBP-9 originated under salt stress conditions, showed improved plant growth, such as increased shoot and root length, along with balanced chlorophyll content compared to controls (Singh and Jha, 2017). Similar results were reported with another strain of *S. maltophilia* BJ01 by Alexander et al. (2020), they found that peanut crop showed improved growth and enhanced photosynthetic pigments and growth hormones under salt stress conditions. In a recent study, Nigam et al. (2022) reported that *Stenotrophomonas* sp. were effective in increased growth, protein accumulation, osmotic adjustment, and Ascorbate peroxidase (PAX) activity in soybean and spinach cultivars under salt stress conditions.

In another study, Bashandy et al. (2020) isolated *S. maltophilia*-SR1 from oil free soils and applied it to soil contaminated by oily wastewater, reporting that the strain successfully used several aromatic hydrocarbons, including benzene, toluene, and xylene, as its sole carbon source and showed plant growth promoting (PGP) properties (indoleacetic acid (IAA), and phosphate solubilization). The mode of action of the arsenic-resistant *S. maltophilia* S255 isolated by Huda et al. (2022) appears to involve several mechanisms such as auxin and hydrogen cyanide production, phosphate solubilization, and nitrogen fixation. These mechanisms may enhance plant growth and improve nutrient uptake, which could be beneficial in agriculture. However, it is important to note that these findings were obtained from a glasshouse study, and further research is needed to confirm the efficacy of this bacterium in large multilocated field trials. It is possible that the efficacy of the arsenic-resistant *S. maltophilia* S255 could vary depending on the origin of the isolates and that native isolates should be considered for recommendation. Therefore, future research should focus on investigating the efficacy of this bacterium in different regions and under various environmental conditions. It is also important to evaluate the safety and potential risks associated with the use of this bacterium as a growth promoter in agriculture.

### *Stenotrophomonas* in nitrogen fixation

Nitrogen-fixing microorganisms use a complex enzyme system called nitrogenase to convert atmospheric elemental nitrogen into plant-usable forms (Masson-Boivin and Sachs, 2018). Nonsymbiotic nitrogen fixation occurs among various genera, including *Acetobacter, Arthrobacter*, *Azotobacter, Bacillus, Clostridium, Diazotrophicus, Pseudomonas,* and *Stenotrophomonas* (Fitton et al., 2019), while symbiotic nitrogen fixation occurs among the members of Rhizobiaceae family along with leguminous plants (Dinnage et al., 2019). Beneficial soil microorganisms, including PGPR, are responsible for fixing a large portion of the elemental nitrogen that enters the soil under natural conditions (Tang et al., 2020). Thus, biological nitrogen fixation via plant-microbe interactions is a major factor in the manufacturing of organic fertilizers (Singh et al., 2023).

Recent research has shown that the *S. maltophilia* strain UPMKH2 can increase rice yield and productivity by providing the plant with a steady supply of nitrogen (Badri Fariman et al., 2022). As a nitrogen source, these bacteria can produce plant growth regulators like IAA, and ACC deaminase (precursor of ethylene), which aid plants in absorbing nutrients and expanding their tissues (Sarkar et al., 2018). In addition to maize, peanuts, rice, sugarcane, and wheat and have all been shown to benefit from *S. maltophilia’s* nitrogen-fixing abilities (Wang et al., 2018a) as PGPR. Different PGP-traits and nitrogenase activities were confirmed with nitrogenase (*nifH*) gene amplification after strains of *S. maltophilia*-COA2 were selected and identified by sequencing their 16S rRNA gene.

To combat sugarcane diseases and cut down on nitrogen fertilizer use, researchers have looked into *S. maltophilia* -COA2 for the first time (Singh et al., 2020). Cerezer et al. (2014) evaluated the nitrogen-fixing capabilities of the atmosphere and symptomatic *Stenotrophomonas* spp. It was discovered that *Stenotrophomonas* isolates have the ability to fix atmospheric nitrogen in the soil, leading researchers to speculate that the reduction of *nifH* clusters of genes is a conservation of energy adaptation of *Stenotrophomonas* during its evolution from a free-living to an opportunistic pathogenic form. Inoculating foxtail millet with the nitrogen-fixing strain *S. rhizophila* EU-FEN-32 as part of a microbial consortium resulted in greater increases in growth and physiological parameters compared to both synthetic fertilizer and the untreated control (Kaur et al., 2023).

Endophytic association with *S. maltophilia* is beneficial to anti-fungal activity and plant growth (phytohormone induction, N2 fixation) (Rojas-Solís et al., 2018a). In addition, *S. pavanii*, a Gram-negative, non-motile, and spore-less species, fixes N_2_ in sugarcane (Ramos et al., 2011). Recent research has shown that common nitrogen fixers, such as rhizobia, do not always colonize or infect the plant roots of leguminous plants but instead typically coexist with *Stenotrophomonas* in other plants. Synergistic processes for nodule formation and enhanced nitrogen fixation capabilities have been postulated when PGPR, like *Stenotrophomonas* species, interact with *Rhizobium* (Abd-Alla et al., 2019). The ability to form nodules in the roots of *Robinia pseudoacacia* has been attributed in part to the horizontal transfer of essential nodulation and nitrogen-fixation genes from rhizobia to other Gammaproteobacteria (*Stenotrophomonas*) and Betaproteobacteria (*Burkholderia*) (Abbott and Peleg, 2015). These nitrogen-fixing *Stenotrophomonas* species can be evaluated in other cropping systems for their potential role in nitrogen fixation and any other side effects on beneficial microbes found in the soil ecosystem.

### *Stenotrophomonas* in phosphorous solubilization

One of the main macroelements that plants require for growth and development is phosphorous, but due to its poor solubility, most of the phosphorous in the soil is unreachable to plants. Phosphorous cannot be utilized by plants because it quickly precipitates in the soil as insoluble combinations with a variety of cations, including Mg, Ca, Al, and Fe. Because P ions are strong ligands, they frequently unite with metal ions to create complexes (Shen et al., 2011). Despite their numerous disadvantages, chemical phosphorous fertilizers are frequently recommended for agricultural soils with a phosphorous deficiency. Due to a confluence of environmental factors and the excessive use of synthetic fertilizers, soil fertility is declining (Ali et al., 2021).

Phosphorus-solubilizing microorganisms are being investigated as an alternative way to address these issues and meet the phosphorous requirements of crop plants. Although many phosphorous solubilizing microorganisms have been identified (Kishore et al., 2015), the majority of them are not well suited to the environmental factors that lead to the production of available phosphorous in the field. Similar to this, *Stenotrophomonas* is a potent phosphorus solubilizing bacterial genus that can release an adequate amount of phosphorus in solution from insoluble rock phosphates and calcium phosphate (CP) (Amri et al., 2023). Xiao et al. (2009) used the National Botanical Research Institute Phosphate (NBRIP) medium to isolate *S. maltophilia* YC from Chinese phosphate mines. The isolate efficiently produced 180.5 mg/L when Tricalcium phosphate (TCP) was the only source of soluble P. The medium’s pH decreased from its initial value of 7.0 to its lowest value of 4.3 after 4 days of incubation. According to the high performance liquid chromatography (HPLC) findings, the isolates were gluconic acid producers during the P-solubilization procedure. The isolate was found to solubilize phosphorus most successfully when fed a diet of maltose and ammonium nitrogen, according to the researchers (Paul and Sinha, 2017).

Singh and Jha (2017) also discovered *S. maltophilia* with P-solubilizing potential in the rhizosphere soil of *Sorghum bicolor*. The organism only made a tiny amount of soluble P (10.73 2.34 mg/mL) using TCP. On the other hand, *S. maltophilia* MB9 was discovered to be effective at producing noticeable zones of solubilization on TCP-containing agar plates after being successfully isolated from a marine environment (John and Thangavel, 2017). *Stenotrophomonas maltophilia* AVP27, which was isolated from the chilli rhizosphere, produced significant zones of P solubilization (Kumar and Audipudi, 2015). TCP significantly increased the isolate’s ability to produce soluble P, as determined by quantitative techniques. *Stenotrophomonas maltophilia* MTP 42 was found to produce 362 mg/mL of soluble P in the rhizosphere soil of *Coleus forskohlii* (Patel and Saraf, 2017). It has been found that *Stenotrophomonas* sp. RC5 was discovered in the rhizosphere of ray grass (*Lolium perenne*), where it synthesizes carboxyl and hydroxyl ions to chelate cations or lower the pH to release P. In the periplasm, the direct oxidation path produces the organic acids. P ions are released due to the substitution of H^+^ for Ca^2+^ that occurs during the excretion of these organic acids, which acidifies the microbial cells and the surrounding environment (Barra et al., 2019). These *Stenotrophomonas* species can be used in various crops for phosphate solubilization and plant growth improvement, but further research is needed to determine any side effects.

### *Stenotrophomonas* in potassium solubilization

Although nitrogen and phosphorus have been extensively studied in relation to the success of exotic species invasions (Mudau et al., 2007; Sinha and Tandon, 2020), the role of potassium in such invasions has received much less attention. However, K is the second most abundant nutrient in leaves, after nitrogen, and the most abundant cation in plant cells. Soil is home to a wide range of potassium-solubilizing bacteria (KSB), as has been demonstrated by numerous studies (Han and Lee, 2005; Kumar et al., 2015; Meena et al., 2015; Sun et al., 2020). *Bacillus, Pantoea, Paenibacillus, Pseudomonas, Rahnella,* and *Stenotrophomonas*, are some of the KSB genera that have been the subject of research in the past (Bahadur et al., 2019; Adeleke and Babalola, 2022). *Stenotrophomonas maltophilia* MB1, MB5, MB6, and MB9 potassium solubilization and biocontrol activities like production of ACC deaminase, siderophore, and yield enhancing strains are isolated from marine environments, in which MB9 is the most potent and dominant strain after application of *S. maltophilia* MB1, MB5, MB6, and MB9 in crops (John and Thangavel, 2017).

In another study, *S. maltophilia* RSD6 has been successfully isolated from the rhizospheric soil of *Oryza sativa*, and can be used as an alternative to agrochemicals (Nevita et al., 2018). *Bacillus* spp., *Burkholderia* spp., *Pseudomonas* spp., and *Stenotrophomonas* spp., were all isolated from tea rhizosphere soil and proven to be effective KSB strains (Gopi et al., 2020). Only *Streptomyces alboviridis, S. rhizophila,* and *Nocardiopsis alba* out of a panel of *Actinobacteria* strains studied were able to dissolve potassium from mica. By Pérez-Martínez et al. (2020) 15 *Stenotrophomonas* strains were isolated from the maize rhizosphere, and two of these were able to soluble potassium sources, while another six showed antagonisms against the pathogen. Acidic and neutral pH usually led to more K release, whereas alkalinity conditions only made *Stenotrophomonas* sp. INCA-FRr1 release more K (Verma et al., 2016). These *Stenotrophomonas* species can be utilized in food crops to enhance crop growth after multilocation and large-scale field trials, however, more potential KSB strains of *Stenotrophomonas* that have both disease control and plant growth promotion activity should be identified in future research so that they could be used as multi-fold agents.

### *Stenotrophomonas* in phytohormones production

Apical dominance, cell elongation, cell division, tissue differentiation, and intracellular communication are just some of the physiological processes that are influenced by phytohormones, also known as plant growth regulators, which are substances synthesized by plants and act as signaling molecules (Tshikhudo et al., 2023). There are five broad categories based on their structural make-up and how they interact with plants’ physiological processes. Auxins, gibberellins, cytokinins, ethylene, and abscisic acid make up the big five. In order to combat the harmful effects of environmental stress, plants will often keep their levels of endogenous hormones constant (Kumar et al., 2012). Phytohormones are produced by a diverse group of bacteria found in plants and soil. Plants’ responses to hormones are crucial to their development and growth. Phytohormones play a significant role in mitigating both biotic and abiotic stress. Plant growth is controlled by a variety of hormones, including gibberellins, auxins, and cytokinins, and these hormones have been linked to developmental processes in plants (Wani et al., 2016). Here we describe how *Stenotrophomonas* affects plant growth and development. Gibberellic acid (GA), ethylene, and indole acetic acid (IAA) were the plant growth regulator traits found in *S. maltophilia* (Singh and Jha, 2017). Deconjugation of gibberellin-glucosyl conjugates secreted from the roots stimulates plant growth (Jędrzejuk et al., 2023). Roots contain inactive 3-deoxy gibberellins, which are converted by bacteria and fungi into their active forms, GA1, GA3, and GA4 (Salazar-Cerezo et al., 2018).

Many aspects of plant development, such as differentiation and cell division, organogenesis, tropic responses, and gene regulation, are controlled by plant growth regulators like IAA (Ryu and Patten, 2008). Many different rhizobacterial strains have been shown to significantly increase plant growth by producing IAA. Due to their consistent release of IAA at minimal concentrations, some strains are also considered to be particularly effective at accelerating plant growth (Tsavkelova et al., 2007). Plant-associated *Stenotrophomonas* species, like many others, were found to effectively produce IAA in the medium used for crop cultivation, with or without the addition of tryptophan. IAA production in 16 *Stenotrophomonas* isolates (both clinical and environmental) was studied by Suckstorff and Berg (2003) and every single isolate tested positive for IAA production.

A study conducted in Germany with *S. maltophilia* e-p19 had an IAA concentration of 5.2 mg/mL, while *S. maltophilia* e-a23 had a concentration of 0.7 mg/mL. Isolates found to be part of environmental clusters were also found to produce more IAA than clinical cluster isolates (Hassan and Bano, 2016). In another study, IAA was found to be produced by *S. maltophilia* BE-25, which was isolated from the root of a banana plant. With or without tryptophan, IAA production from the isolate was sufficient, according to a thin layer and high-performance liquid chromatography analysis (Ambawade and Pathade, 2013). *Stenotrophomonas maltophilia*, isolated from the forest soil, also produced IAA in higher amounts (50.4 ± 0.9 g/mL) (Amri et al., 2023). The isolate was also capable of producing gibberellic acid, another plant growth regulator. *Stenotrophomonas maltophilia,* obtained from the rhizosphere of *Cenchrus ciliaris*, was also studied for its ability to promote plant development in the presence of tryptophan under salt stress conditions (Hassan and Bano, 2016). Their findings suggest that *S. maltophilia* IAA production is crucial to the induction of salt tolerance. However, their actual efficacy on a large field scale requires further attention for their commercialization.

### *Stenotrophomonas* in siderophore production

Due to its importance in respiration, DNA synthesis, heme formation, and other biochemical reactions, iron deficiency can stunt the development of plants (Shetty and Corson, 2020). Iron is present in high concentrations in the Earth’s crust, but its bioavailability is limited due to the insoluble nature of the Fe^3+^ ion (Behnke and LaRoche, 2020). Common minerals’ hydroxide and oxide phases tend to accumulate iron, rendering it unavailable to plants and other living things. *Stenotrophomonas* is one of the PGPR that can make a siderophore, which is utilized to remove iron from insoluble mineral phases. According to their chemical makeup and coordination site, these low molecular weight (500–1,000 Da) ferric ion chelating compounds can be grouped into three groups: the catecholate, the carboxylic type, and the hydroxamate type (Pan et al., 2022). Although many pathogenic organisms view the production of siderophores as a virulence factor, plant-associated organisms view it as a growth-promoting trait because it facilitates iron uptake and prevents the spread of plant pathogenic microorganisms.

In addition to PGPR strains, it has been reported that several plant-associated *Stenotrophomonas* strains also produce siderophores. Like many other strains of *S. maltophilia,* SPB-9 was found to produce an orange color zone on chrome azurol S (CAS) agar plates (Singh and Jha, 2017). Hydroxamate-type siderophores are produced by *S. maltophilia* MTP-42, which has been found in the rhizosphere of *Coleus forskohlii* (Patel and Saraf, 2017). Ghavami et al. (2017) studied the siderophore production by *S. chelatiphaga* LPM-5 T and reported that in the CAS liquid medium, the isolate was found to produce 12.66% siderophore. In a spectrophotometric test performed by Wilson et al. (2016), positive results indicated that the isolate generated a carboxylic siderophore. Siderophores are essential for preventing the invasion of pathogenic fungi and also help plants absorb more iron. By limiting the amount of ferric ions available, the growth of pathogenic fungal strains is stifled (Kloepper et al., 1980). Several strains of *Stenotrophomonas* were found to have the ability to produce siderophores, which were then shown to inhibit the growth of pathogenic fungi. *Stenotrophomonas* can scavenge molecules of the siderophore class that are produced by other microorganisms, such as the phytopathogenic fungus *Ustilago* (Sun et al., 2022). TonB-dependent outer membrane protein receptors (TBDRs) have been reported to be present in the genome of *S. maltophilia*, and their primary function is the active transport of the iron-siderophore complex. This superior iron uptake capacity suggested that they could pose a threat to other organisms as they evolved into endophytes or rhizosphere residents.

### Exopolysaccharides (EPS) production by *Stenotrophomonas* spp.

Bacterial EPS supplies protection from different environmental stresses, such as predation, desiccation, and the effects of antibiotics (Limoli et al., 2015). EPSs have an important role in the aggregation of bacterial cells and supply carbon when the substrate is in low concentration (Banerjee et al., 2021). The biosynthesis of EPS by bacterial cells depends upon environmental and nutritional conditions (Nouha et al., 2018). Different microorganisms utilize various sources of carbon and nitrogen and differ in their mineral requirements, pH, and temperature, which are important factors for maximum EPS production (Aeron et al., 2020). *Brevibacillus parabrevis* (V4) and *S. maltophilia* (c6) were the two nodule endophytic isolates with the highest EPS production capability (among C1-C13 and V1-V7) (Abd-Alla et al., 2018). This research suggests that *Brevibacillus parabrevis* (V4) and *S. maltophilia* (c6) can generate a high yield of EPS when fed a diet rich in sucrose. *Stenotrophomonas maltophilia* (c6) does not use date molasses, lactose, galactose, or glucose as carbon sources for EPS production. Potassium nitrate stimulated EPS production in *S. maltophilia* (c6) and glycine did the same for *B. parabrevis* (V4) (Castellane et al., 2015). It is worth noting that *S. maltophilia* (c6) EPS yield and growth are suppressed by Fe_3_O_4_ (25–200 g/mL) and Fe_2_O_3_ (20–100 g/mL) NPs at varying concentrations. *Stenotrophomonas maltophilia* strain WR-C isolated from a clogged septic tank system that consistently formed biofilms on sand grains produced EPS, and caused clogging in the sand column (Abd-Alla et al., 2018).

### *Stenotrophomonas* in rhizoremediation

Plant enzymes initiate the degradation of substances during phytoremediation, while the local microbial population carries it out during natural attenuation or bioaugmentation. It has been reported in several studies that certain microbes in the rhizosphere of plants used for phytoremediation or of plants growing from surrounding vegetation on a contaminated site play a significant role in the degradation of pollutants. This process, known as rhizoremediation, involves the rhizobacterial community (Rane et al., 2022). Sometimes, bacteria in the rhizosphere are essential to the decomposition process. There is widespread agreement that microbial bioremediation processes are a useful method for cleaning up polluted areas. This catabolic plasticity plays a crucial role in the breakdown of xenobiotic compounds and the conversion or accumulation of environmental pollutants.

In order to understand metabolic and regulatory networks and to provide novel pathways and microorganisms that will be useful for future applications, genome-based global studies are on the rise. After analyzing their genomes, scientists discovered that different strains of *Stenotrophomonas* can produce enzymes that aid in the breakdown of polychlorinated hydrocarbons and metals. Significant roles for *Stenotrophomonas* spp. in the breakdown of geosmin, hexahydro-1, 3, 5-trinitro-1, 3, 5–triazine (RDX), keratin, macrocyclic hydrocarbon, nitrophenol, and phenanthrene (Gao et al., 2013). Species of *Stenotrophomonas* have been found effective for bioremediation of agricultural soil by removing various chemical pesticides and insecticides, in addition to a wide range of environmental pollutants. The species will be useful in reducing the need for harmful chemicals in farms. A strain of *S. acidaminiphila* isolated by (Uniyal et al., 2016) from the rhizosphere soil of *Zea mays* was found to degrade fipronil, a common insecticide. A novel fipronil degrading pathway for the isolate has been proposed, and it has been suggested that the isolate could be used for bioremediation of fipronil-contaminated soil. Pourbabaee et al. (2017) also reported that *S. maltophilia* degraded diazinon (a pesticide). They also provided an analysis of the pesticide’s likely degradation pathway based on FTIR. A mineralization pathway involving dechlorination, hydroxylation, and carboxylation processes was proposed based on genome annotation of the DDT degradation gene and GC–MS analysis of metabolites (Pan et al., 2016).

Zaffar et al. (2018) reported that a *S. maltophilia* EM-1 strain degrades the organochlorinated pesticide endosulfan. Endosulfan was the only sulphur source used by the bacteria. According to gas chromatography mass spectrometry testing, the bacteria are capable of metabolizing endosulfan into safer compounds like endosulfan diol. Organophosphorus insecticides belonging to the groups O, O-dialkyl phosphate, and O, O-dialkyl phosphorothioate were degraded by *Stenotrophomonas* sp. G1, which was isolated from the sludge of a chlorpyrifos manufacturer plant (Deng et al., 2015). Two *Stenotrophomonas* isolates, *S. maltophilia* MHF ENV20 and *S. maltophilia* MHF ENV23, were found to degrade chlorpyrifos, cypermethrin, and fenvalerate (Fulekar, 2014). It has also been reported that *S. maltophilia* M1 degrades the nemato-pesticide methomyl (Oxime carbamates). The strain was discovered at a site where methomyl was used for irrigation. A 5 kb plasmid (PMb) containing the gene responsible for methomyl degradation was confirmed after the transformation of the plasmid DNA into *Escherichia coli* (Mohamed, 2009).

In a symbiotic association with strain *Stenotrophomonas* sp. W16, the rate at which fomesafen is degraded in the soil increases from 29.17 to 57.87%. To aid in the bioremediation of herbicide-contaminated farming soil, this study presents a novel fomesafen-degrading rhizobium that may be used in conjunction with legumes in symbiotic systems to break down the chemical (Chen et al., 2023). Pesticides often have residual concentrations that are above regulatory thresholds. Wherever this is a problem, getting chemical-free agricultural soils fit for growing eco-friendly crops is a major obstacle. Since bioremediation is a greener, cheaper, and more effective method than physical and chemical methods, it can be used to exploit the microbial metabolism of native microorganisms for degradation.

## Potential risks and challenges of using *Stenotrophomonas* spp. in agriculture

*Stenotrophomonas* is a novel, multi-antibiotic resistant, opportunistic plant-associated bacterium on a global scale. It is reported that this bacterium remains associated with several plants. Moreover, it is used in bioremediation techniques and sustainable agricultural practices as a microbial biocontrol or anti-stress agent for crops. Several studies have demonstrated the immense potential of *Stenotrophomonas* spp. in agriculture; they may enhance plant growth and germination while suppressing plant diseases (Messiha et al., 2007).

On the other hand, *S. maltophilia* is also a novel human pathogen that can cause infectious diseases in humans. The only *Stenotrophomonas* species known to cause human illness is *S. maltophilia*; however, isolates of this species vary widely in terms of phylogeny and phenotype (Roscetto et al., 2008). This is probably related to the plethora of environmental niches occupied by this bacterium; the vast majority of infections are likely due to contact with distinct environmental sources. In fact, *S. maltophilia* epidemics are uncommon and are induced by contaminated origin, such as water sources (Park et al., 2008). *S. maltophilia* strains are undoubtedly equally capable of infecting people but only affect those with underlying illnesses, not the general population (Lira et al., 2017). The innate resistance of *S. maltophilia* to many first-line antimicrobials, including beta-lactams, macrolides, tetracycline, chloramphenicol, and quinolones, is the primary cause of the increase in *S. maltophilia* infections (Crossman et al., 2008). Additionally, *S. maltophilia* isolates may quickly evolve resistance to newer antibiotics via mutation; the underlying processes are unknown but are likely to be the consequence of excess production of intrinsic efflux transporters (Gould and Avison, 2006). Insufficient information exists regarding the pathogenicity of these organisms to humans. It is further demonstrated by the phenotypic and phylogenetic diversity analysis that there is a difference between pathogenic and other strains, but it is unquestionably a PGPR. Field tests and other direct applications cannot be conducted with opportunistic pathogens.

However, since *S. rhizophila* is not recognized as a human pathogen, these strains are interesting choices for biological control and stress resistance in plants because they often live endophytically. *Stenotrophomonas pavanii* is a recently discovered bacterium that can fix nitrogen from the atmosphere. The *S. pavanii* strain obtained from sugar cane is widely used in organic agriculture (Ramos et al., 2011). *S. pavanii* is a unique species of PGPR, and it is thought to focus on improving development and yield in many crops.

The diversity of *Stenotrophomonas* strains makes it difficult to distinguish characteristics because of their advantageous interactions with plants and their facultatively harmful infections with humans (Berg and Martinez, 2015). Differentiation of characteristics is crucial for future agricultural applications as well as for our understanding of infection risks and associated epidemiological issues. Predicting beneficial pathogen threats to human health is one of the current difficulties for agricultural biotechnology. Therefore, there is room to develop a new aspect of *Stenotrophomonas* spp. uses and importance in agriculture.

## Future remarks

The genus *Stenotrophomonas* has been shown to possess a number of functional properties, and the research performed so far has demonstrated that it can be used to manage pests and pathogens as well as promote plant growth. However, there are still gaps in research that must be filled. For example, few species of *Stenotrophomonas* are human pathogens that can cause serious diseases in humans; however, further research is needed to discriminate between human pathogenic and non-pathogenic strains of *Stenotrophomonas* spp. and their deployment in crop improvement and *ex-situ* conservation. Using the novel molecular tools, the human pathogenic strains of *Stenotrophomonas* can be engineered into non-pathogenic strains so that they could be used in food crops for growth enhancement as well as for management of pests and diseases.

Although many studies were conducted in the past on exploring the applications of *Stenotrophomonas* in crop protection and crop improvement, further investigations are required to explore the various functions of *Stenotrophomonas* in plant growth promotion under diverse growth conditions and to demonstrate their interaction with the plant and soil systems under various environmental conditions. Further, advanced molecular techniques should be developed to identify and discriminate between human pathogenic and non-pathogenic strains of the genus *Stenotrophomonas,* so that non-pathogenic strains can be deployed in diversified cropping systems. Besides, molecular mechanisms underlying *Stenotrophomonas-*plant interactions are poorly understood and need to be carried out using advanced omics approaches such as transcriptomics, proteomics, and metabolomics. Further, as an opportunistic mycoparasite, research into its induction and regulation of enzyme expression is needed in order to improve its biocontrol abilities and to come up with potential commercial bio-fungicides. Besides, identifying various physiological traits to enhance industrial application of *Stenotrophomonas* as an alternative strategy for producing antibiotics and enzymes could be other areas for future research.

## Conclusion

In conclusion, the use of beneficial microorganisms for crop health management has been studied in many cultivated crops. In this regard, *Stenotrophomonas* species have been proven to be efficient microbial biocontrol agents and have exhibited both antagonistic activity against phytopathogens and entomopathogenic activity against insect pests. The biocontrol capability of *Stenotrophomonas* spp. includes both chitinolytic and proteolytic activities, which may protect plants against phytopathogens but also play an important role in activating plant defense systems. In particular, research conducted worldwide demonstrates that various *Stenotrophomonas* species are effective in managing many pests and diseases of food crops, as well as promoting plant growth. The potential strains of *Stenotrophomonas* spp. for crop health improvement identified by the researchers can be commercialized among farmers after their validation under field conditions in larger scale/multilocation trials. However, more work is needed to determine the cost–benefit ratio for economically commercializing bio-fertilizers and biopesticides based on *Stenotrophomonas* species. Furthermore, *Stenotrophomonas* species-based bio-products must be evaluated for regulatory risk parameters before they can be given to the farmers.

## Author contributions

AK, LR, and VK: conceptualization and writing—original draft preparation. AKP and NR: writing—review and editing. KC and A: review and editing. All authors contributed to the article and approved the submitted version.

## Funding

AKP received funding from Department of Science and Technology (DST), Science and Engineering Research Board, Government of India (SRG/2021/000299) through Start-up Research Grant.

## Conflict of interest

The authors declare that the research was conducted in the absence of any commercial or financial relationships that could be construed as a potential conflict of interest.

## Publisher’s note

All claims expressed in this article are solely those of the authors and do not necessarily represent those of their affiliated organizations, or those of the publisher, the editors and the reviewers. Any product that may be evaluated in this article, or claim that may be made by its manufacturer, is not guaranteed or endorsed by the publisher.
